# The Effect of Reward Magnitude on Different Types of Exploration in Human Reinforcement Learning

**DOI:** 10.1007/s42113-024-00224-6

**Published:** 2024-10-03

**Authors:** Kanji Shimomura, Kenji Morita

**Affiliations:** 1 Graduate School of Education, The University of Tokyo, Tokyo, Japan; 2 Japan Society for the Promotion of Science, Tokyo, Japan; 3 International Research Center for Neurointelligence (WPI-IRCN), The University of Tokyo, Tokyo, Japan

**Keywords:** Reinforcement learning, Reward magnitude, Random exploration, Directed exploration, Explore-exploit tradeoff

## Abstract

How humans resolve the explore–exploit dilemma in complex environments is an important open question. Previous studies suggested that environmental richness may affect the degree of exploration in a type-specific manner and reduce random exploration while increasing uncertainty-based exploration. Our study examined this possibility by extending a recently developed two-armed bandit task that can dissociate the uncertainty and novelty of stimuli. To extract the pure effect of environmental richness, we manipulated the reward by its magnitude, not its probability, across blocks because reward probability affects outcome controllability. Participants (*N* = 198) demonstrated increased optimal choices when the relative reward magnitude was higher. A behavioral analysis with computational modeling revealed that a higher reward magnitude reduced the degree of random exploration but had little effect on the degree of uncertainty- and novelty-based exploration. These results suggest that humans modulate their degree of random exploration depending on the relative level of environmental richness. Combined with findings from previous studies, our findings indicate the possibility that outcome controllability also influences the exploration–exploitation balance in human reinforcement learning.

**Supplementary Information:**

The online version contains supplementary material available at 10.1007/s42113-024-00224-6.

## Introduction

A reinforcement learning agent faces exploration–exploitation tradeoff when it aims to maximize future reward (Sutton & Barto, 2018). Exploiting the well-known option that is estimated to be the best can offer a high reward, but repeated exploitation may hide potentially better options from the agent. To avoid this problem, the agent needs to explore alternatives to identify those that may eventually lead to the highest reward. However, in doing so, it risks losing other opportunities to exploit. An important open question is how humans address this explore-exploit tradeoff.

It has been suggested that humans adopt two types of exploration strategies: random and directed exploration (Wilson et al., 2014). Random exploration occurs by chance and is driven by the noise of the decision-making system. Conversely, directed exploration is actively aimed at obtaining information about a reward space. Directed exploration is computationally realized by adding “information bias” to the value of the options. Humans possibly use both uncertainty (e.g., Trudel et al., 2021; Wiehler et al., 2021; Wu et al., 2018) and novelty (e.g., Costa et al., 2019; Wittmann et al., 2008) of the options to guide directed exploration.

In most cases, uncertainty and novelty are correlated; novel options are maximally uncertain and certain options are highly familiar to the agent. Imagine a situation in which a new restaurant opens in your town. At first, the restaurant is visually novel to you and it would also be highly uncertain because you have no information about the quality of its dishes or service. However, as you visit the restaurant several times, you gain knowledge about the menu and service, thus reducing uncertainty. At the same time, you inevitably become accustomed to the restaurant, which leads to a decrease in its novelty. This correlation renders it difficult to distinctly examine the effects of uncertainty and novelty on exploration. However, one recent study developed a new two-armed bandit task that allowed for separating the uncertainty and novelty of options and found that both impacted decision-making differently (Cockburn et al., 2022). Specifically, while uncertainty had a time-dependent effect on choice, which decreased, rather than increased, exploration as the task approached its end, novelty constantly enhanced exploration by acting as a positive bias toward utility and suppressing the effect of uncertainty (Cockburn et al., 2022). This suggests that dissociating these two elements when examining directed exploration is desirable.

Several psychological factors have been shown to impact the degree of random and/or directed exploration. For instance, Brown et al. (2022) showed that, while an increase in environment size led to a reduction in both random and directed exploration, an increase in memory load decreased directed exploration but increased random exploration. Also, an individual’s dopamine level (Chakroun et al., 2020) and motivational state (Sinclair et al., 2023) were shown to affect directed exploration but not random exploration. These findings indicate that humans adaptively modulate the balance between exploration and exploitation, often in a type-selective manner, depending on their states or current environment.

Another possible environmental or state-like factor that influences the explore-exploit balance is the average level of reward expectancy, that is, how much reward can be expected on average in an environment. Using reinforcement learning and foraging tasks, several studies have suggested that such “environmental richness” can cause changes in learning and decision-making in mice (Ohta et al., 2021) and humans (Constantino & Daw, 2015; Teodorescu & Erev, 2014), which could alter the explore-exploit balance by, for instance, stimulating more positively biased learning rates (Ohta et al., 2021) and an increase in harvesting behavior (Constantino & Daw, 2015). Given that several factors selectively influence random or directed exploration, it is possible that environmental richness exerts distinct effects on different types of exploration. Specifically, in relatively rich environments, random exploration is expected to decrease so that rewards can be earned in a greedy manner. However, to obtain information that may be useful in subsequent opportunities, an increase in uncertainty-based exploration is likely, especially when many opportunities for rewards remain (Wilson et al., 2014).

Nevertheless, this possibility has not yet been directly tested. In this preregistered study, therefore, we aimed to investigate the effects of environmental richness on different types of exploration in humans. To this end, we adopted the novel two-armed bandit task (Cockburn et al., 2022; Nussenbaum et al., 2023), which can separate the uncertainty and novelty of options, with a modification that enabled us to manipulate the richness of rewards. Some previous studies (Ohta et al., 2021; Teodorescu & Erev, 2014) have manipulated rewards by their probability, which affects the perceived controllability of the outcomes (Maier & Seligman, 1976) and has been used to manipulate controllability (Dorfman & Gershman, 2019). Given that controllability is a core aspect of learned helplessness (Maier & Seligman, 2016), in which an agent demonstrates a lack of exploration and deficits in learning in a new environment, controllability is likely to have a confounding effect on exploration. Therefore, in this study, we manipulated rewards not by the reward probability of options but by the reward magnitude of blocks and compared the behavior of participants in high and low reward blocks. Based on the possibility that, as mentioned earlier, environmental richness differentially affects random and directed exploration, we hypothesized that, when the relative reward magnitude is higher, participants will show (1) less random exploration and (2) more uncertainty-based exploration. We did not construct a specific hypothesis for novelty-based exploration due to limited prior research on the issue.

## Methods

### Participants

Two hundred participants with a mean age of 22.95 years (SD = 3.35 years; range, 18–38 years, 97 female, 100 male, 1 other, 1 no answer) completed the experiment. Participants were recruited via a website (https://www.jikken-baito.com) that provides information on participation in psychological experiments and surveys in Japan. We determined the sample size to detect a small effect size for the possible within-participant differences in the estimated parameters of computational models between the high reward and low reward conditions. Although we took a different approach as a result of the revision (see “Bayesian Reinforcement Learning Modeling” section for details), in the pre-registered analysis (https://osf.io/tjba9/), we originally planned to conduct model fitting separately for the two conditions and compare the differences in parameters using a paired *t*-test. Using G*power 3.1 (Faul et al., 2009) and assuming a small effect size for a paired *t*-test of *d* = 0.2 according to Cohen (1988), we obtained a required sample size of 199 with an *α* of 0.05 and a power of 0.8. The number of participants included in the analysis for the main task was 198, because two participants (both male) were excluded due to their poor performance in a memory test (accuracy below 60%; see “Memory Test” section).

The research ethics committee at the University of Tokyo approved the procedures before starting the study (23–269). Informed consent was obtained from all participants (for details, see Consent to participate section). All participants were paid 2100 yen (about 14.2 dollars) for the experiment, which lasted for about 1.5 h. In addition to this baseline payment, participants were told that they could receive an additional bonus payment based on their performance in the experiment (see the “Two-Armed Bandit Task with Bonus Slot Machines” section for detailed instructions on the calculation of the bonus). In reality, participants uniformly received bonus payment of 646 yen (about 4.4 dollars), which was determined by the median performance of all participants.

### Procedure

The experiment was conducted online using jsPsych (de Leeuw et al., 2023) and DataPipe (de Leeuw, 2023). Participants first answered a questionnaire that included scales to measure state optimism and depressive symptoms. This questionnaire was included in the procedure to examine the association between state optimism or depressive symptoms and behavior in the two-armed bandit task, which is not the focus of this paper. Results regarding these individual differences will be reported elsewhere.

After finishing the questionnaire, participants completed the two-armed bandit task. Before proceeding to the main trials, participants completed 10 practice trials and received instructions about the task. Next, they answered five questions about the content and rules of the task. Participants could start the main trials only after they correctly answered all five questions. After completing the two-armed bandit task, they were asked to take a memory test to check if they appropriately engaged in the task.

### Experimental Design

#### Two-Armed Bandit Task with Bonus Slot Machines

**Fig. 1:**
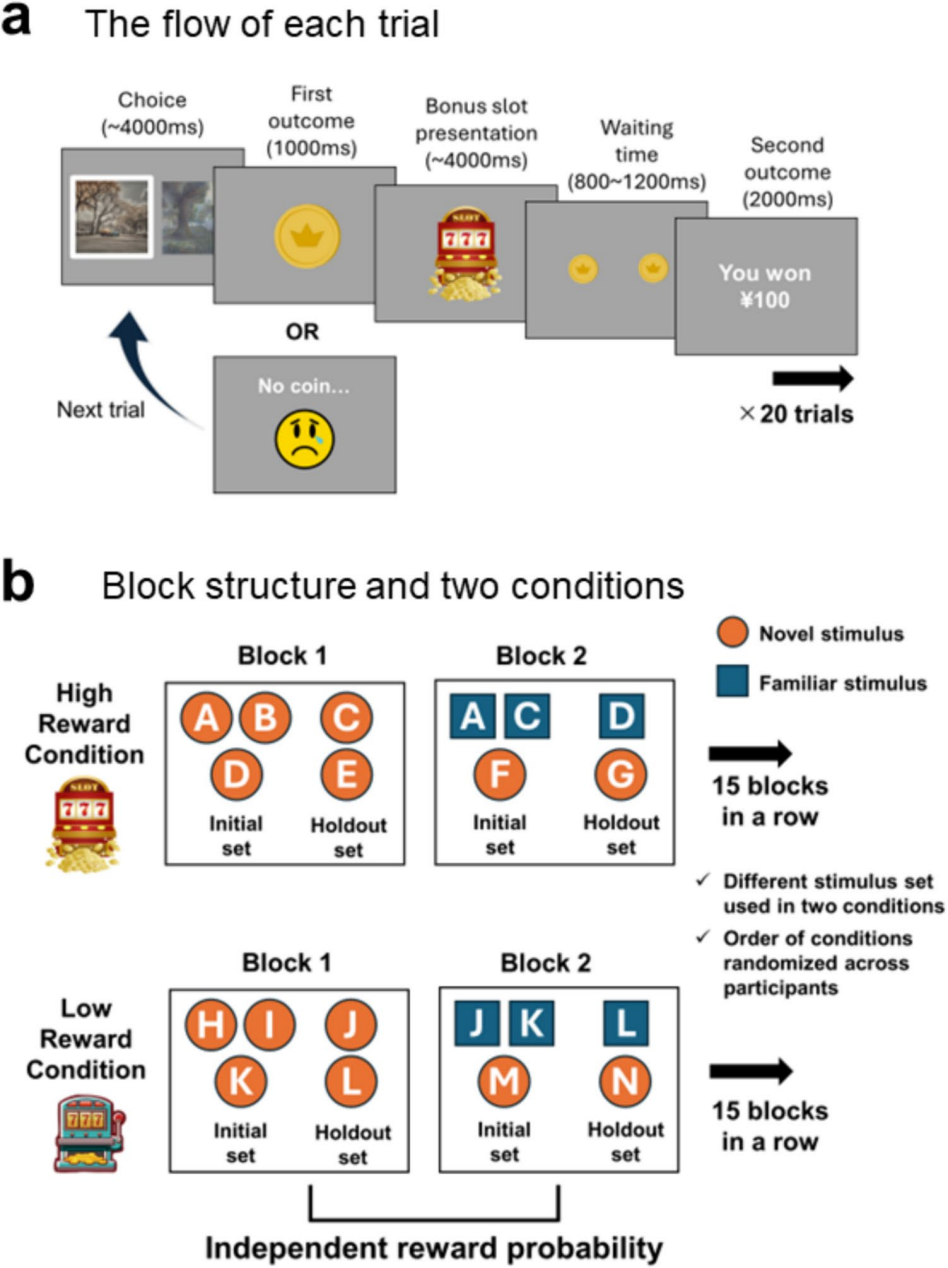
Overview of the two-armed bandit task with bonus slot machines. **a** Trial dynamics. After a 500–1500 ms presentation of fixation, two stimuli were presented in a randomized position (left or right). Participants had to select between the two stimuli within 4000 ms. After the selected stimulus was highlighted for 1000 ms, the first outcome (coin or no coin) was presented for 1000 ms. If participants obtained a coin, they were next presented with a bonus slot machine. Upon pressing a space key, the bonus slot spun for 800–1200 ms, and the second outcome was presented for 2000 ms. **b** Structure of the blocks to manipulate the uncertainty and novelty of stimuli and an overview of the two conditions. Every block had five stimuli consisting of two novel stimuli and three familiar stimuli, which were divided into three initial sets and two holdout sets (see main text for details). The two conditions (high and low reward conditions) consisted of 15 blocks each, and the order of the conditions was pseudo-randomized across participants

The two-armed bandit task in the present study (Fig. 1) is a modified version of the one used in the previous studies (Cockburn et al., 2022; Nussenbaum et al., 2023). The task consisted of 30 blocks of 20 trials each. At each trial (see Fig. 1a), participants had 4 s to select between two options using the “F” or “J” key. If the result of choosing the option was a “loss,” then the trial ended and proceeded to the next trial. When the trial was “win,” on the other hand, participants obtained a coin. Unlike the original design, this coin was not a reward itself (i.e., the coin itself did not affect actual payment), but could be used to spin an additional bonus slot machine. Participants could spin the bonus slot by pressing the space key. If there was no response, the bonus slot machine spun automatically 4 s after its presentation. The bonus slot machine offered a certain amount of positive reward that was determined based on normal distributions.

This bonus slot machine was the novel component added to the original design to manipulate the reward magnitude of each block. Specifically, there were two types of bonus slot machines: rich and normal. The rich bonus slot machine offered rewards based on a normal distribution with a mean of 100 and a standard deviation (SD) of 10, while the reward of the normal slot machine was drawn from a normal distribution with a mean of 20 and an SD of 2. In each block, the available bonus slot machine was fixed to either of these two types. Half of the 30 blocks were assigned to the “high reward condition,” in which the rich bonus slot machine was available, and the other half to the “low reward condition,” in which the normal bonus slot machine was available. Fifteen high reward blocks and 15 low reward blocks were conducted separately (i.e., they were not interleaved), and the order of the conditions (i.e., whether the experiment started with the high reward condition or the low reward condition) was determined randomly across participants (Fig. 1b). Participants were explicitly told, before starting the main trials, about the presence of these two types of bonus slot machines, the specific size of the reward each slot machine was expected to offer (i.e., 100 and 20 for the rich and normal bonus slots, respectively), and the block structure of the task. We informed participants that their bonus payment would be determined by the average reward obtained in the 30 blocks. Therefore, the optimal behavior for participants was to maximize the reward in all the blocks regardless of the type of the bonus slot machine, although the weight for the final bonus payment differed between high reward blocks and low reward blocks.

The two options presented in each trial were selected from five options within that block. The reward probabilities of the five options in each block were randomly sampled from (0.2, 0.35, 0.5, 0.65, 0.8) without replacement. The uncertainty and novelty of each option were systematically manipulated by the structure of the blocks, as was done in the previous study (Fig. 1b; Cockburn et al., 2022). Importantly, among the five options, three were familiar options that had been presented at least four times in the previous blocks, while the remaining two were novel options that had not been shown before. During the early trials within a block, three (two familiar, one novel) out of these five options were used, and the remaining two (one familiar, one novel) were gradually introduced between the 8th and 16th trials, to ensure a clear gradient of novelty and uncertainty throughout the blocks. Another crucial point was that the reward probabilities of the options were independent across blocks, and participants were explicitly told about this structure. This meant that, even for the visually identical options, participants had to relearn their reward probabilities when the block switched. In other words, the uncertainty of the options, but not the novelty, was reset between blocks. This structure allowed for the dissociation of the effects of uncertainty and novelty on choice.

To ensure that all visual stimuli (options) were completely novel to participants, the visual stimuli in this task were generated using the text-to-image model from StabilityAI: “Stable Diffusion 2.1” (https://www.mage.space/) with the following prompt: “an example of an artistic picture.” Different sets of images were used for high reward and low reward conditions, and the stimuli used in each condition were randomized across participants. Specifically, 66 images generated in advance were randomly shuffled and divided into two sets of 33 stimuli for each participant. All images used in the experiment and codes for the experiment are available on https://github.com/Kshimod/MAB.

#### Memory Test

To check if participants engaged in the two-armed bandit task appropriately and if they noticed the novelty of the stimuli, a memory test was conducted after the two-armed bandit task. This test consisted of 60 trials in which participants answered whether they had seen a presented stimulus or not in the main task by pressing the Y (yes) or N (no) key. Half of the 60 stimuli were stimuli that had been presented in the two-armed bandit task (“used stimuli”), and the other half were not. The 30 “used stimuli” consisted of 15 images selected randomly from each of the high reward and low reward conditions. Participants who achieved less than 60% accuracy in this task were excluded from the analysis. Participants included in the analysis (*n* = 198) generally performed well in the memory test (median 93%, min 68%, max 100%), indicating that they focused on the two-armed bandit task and could distinguish between familiar and novel options within the task.

### Statistical Analysis

Analysis was conducted in R version 4.0.3 (R Development Core Team, 2018). Trials with no response (344 trials, 0.0003% of all trials) and with response times shorter than 200 ms (1149 trials, 0.02% of all trials) were excluded from the analysis.

All indices used in the analysis (except for reward magnitude) were defined in the same way as the previous study (Cockburn et al., 2022). The expected win probability of an option ($W({s}_{i})$) was quantified as the mean of a Beta distribution defined according to the number of wins and losses observed within the current block (Beta[$\alpha =$ number of wins + 1; $\beta =$ number of losses + 1]). The uncertainty of each option ($U({s}_{i})$) was defined as the variance of the same Beta distribution. Novelty ($N({s}_{i})$) was defined as the variance of a Beta distribution specified according to the number of times a particular stimulus had been observed across the entire trials (Beta[$\alpha =$ number of exposures + 1; $\beta =$ 1]). Reward magnitude ($M$) was coded as 1 (high) or − 1 (low). All continuous variables except trial number $t$ were standardized into *z*-scores across the entire dataset before inclusion in the models. Trial number $t$ was scaled to the range of 0–1 before being used in the analysis.

The difference in the total number of obtained coins in the two conditions was tested with a paired *t*-test. The optimal choice was defined as selecting the option with the higher expected win probability. The difference in the total number of optimal choices between the high and low reward conditions was tested with a paired Wilcoxon’s signed-rank test because the normality of the difference was rejected according to the result of the Shapiro–Wilk test. The effects of reward probability, uncertainty, and novelty on choice, and their interaction with reward magnitude, were examined with the following mixed-effects logistic regression model, using the “glmer” function of the R package “lme4”:

1 $$p\left({A}_{t}=L\right)=\left({W}_{\Delta }+{U}_{\Delta }+{N}_{\Delta }\right)\cdot{M}+\left(1+\left({W}_{\Delta }+{U}_{\Delta }+{N}_{\Delta }\right)\cdot{M}|ID\right)$$

where $p\left({A}_{t}=L\right)$ represents the probability of selecting the left stimulus at trial $t$, and ${W}_{\Delta }$, ${U}_{\Delta }$, ${N}_{\Delta }$, and $M$ denote the difference between the two presented stimuli in the expected probability of win, uncertainty, novelty, and reward magnitude in the current block, respectively.

The effect of reward magnitude on the interaction between the task horizon and expected win probability, uncertainty, and novelty was explored with the following mixed-effects logistic regression model:

2 $$p\left({A}_{t}=L\right)={W}_{\Delta }\cdot{t}\cdot{M}+{U}_{\Delta }\cdot{t}\cdot{M}+{N}_{\Delta }\cdot{t}\cdot{M}+\left(1|ID\right)$$

where $t$ represents the scaled trial number in the blocks. Note that the model did not include random slopes because estimation did not converge when we used models assuming random slopes. This analysis regarding the effects of task horizon was registered as exploratory analysis in the preregistration document, and thus, we did not have a priori hypotheses about the effects.

### Bayesian Reinforcement Learning Modeling

The logistic regression analysis can quantify the effects of win probability, uncertainty, novelty, and their possible differences between the high and low reward conditions. However, it does not tell us about the specific mechanisms through which those factors affect learning and decision-making. For instance, even if there is a positive effect of novelty on choice, it does not necessarily mean that novel options have a positive bias toward utility. Rather, it might be because the novelty of the options suppresses the negative bias of uncertainty on utility (Nussenbaum et al., 2023). Likewise, even if a positive interaction between expected win probability and reward magnitude is found, several different mechanisms could explain this. It might be due to the difference in the learning process such that participants more accurately learned the values of options in the high reward condition, or due to the difference in the decision-making process such that participants made choices in a greedier way in the high reward condition. Therefore, in order to gain a detailed understanding of the processes underlying the results obtained from the logistic regression, we applied computational modeling to the participants’ behavioral data. We aimed to draw conclusions about the differences in exploration between conditions by integrating the findings from the logistic regression and the computational modeling.

#### Model Description

We fitted six Bayesian reinforcement learning (Bayesian RL) models, which differed in how to calculate utility ($V({s}_{i})$), to the choice data of participants for each condition, as in the previous studies (Cockburn et al., 2022; Nussenbaum et al., 2023), to examine the detailed mechanism of the possible behavioral differences between the two conditions. In these models, each option’s *q*-value (expected win probability) and uncertainty was learned in a similar way as the logistic regression analysis: they were defined as the mean and variance of a Beta distribution specified according to the number of wins and losses within a block. One difference was that in the Bayesian RL models, the agent was assumed to gradually forget the previous observations. Specifically, the two parameters of the Beta distribution characterizing each option $i$ at trial $T$ were represented in the following way:

3 $${\alpha }_{i}=1+\sum\nolimits_{t=0}^{T-1}{\left(1-\eta \right)}^{T-t}\cdot{O}_{t}^{W}$$

4 $${\beta }_{i}=1+\sum\nolimits_{t=0}^{T-1}{\left(1-\eta \right)}^{T-t}\cdot{O}_{t}^{L}$$

where ${O}_{t}^{W}$ and ${O}_{t}^{L}$ are binary flags noting whether or not the observed outcome on trial $t$ was a win or loss, respectively, and $\eta$ represents the forgetting rate parameter. The *q*-values (expected win probability) and uncertainty of each option $i$ were represented by the mean and variance of the Beta distribution, as mentioned above:

5 $$Q\left({s}_{i}\right)=\frac{{\alpha }_{i}}{{\alpha }_{i}+{\beta }_{i}}$$

6 $$U\left({s}_{i}\right)=\frac{{\alpha }_{i}\cdot{\beta }_{i}}{{\left({\alpha }_{i}+{\beta }_{i}\right)}^{2}\cdot\left({\alpha }_{i}+{\beta }_{i}+1\right)}$$

The utility of each option ($V\left({s}_{i}\right)$) was calculated in a different way using these values depending on the models (see below). The agent was assumed to make choices stochastically in a soft-max manner with the calculated utilities of the presented options:

7 $$p\left({c}_{t}=left\right)=\frac{1}{1+\mathrm{exp}\left(\beta\cdot \left({V}_{right}-{V}_{left}\right)\right)}$$

where ${c}_{t}$ is the choice on trial $t$ and an inverse temperature parameter $\beta$ inversely represents the randomness of the choice (choice becomes less random as $\beta$ increases). These were the basic components common to the six candidate models. Specific features of the models were as follows:

1. Baseline model (two parameters): The baseline model calculated utility using only *q*-values (i.e., expected win probability) of options ($Q({s}_{i})$), which were the mean of the abovementioned Beta distribution. Free parameters were $\eta$ and $\beta$.
2. Novelty bias model (three parameters): The novelty bias model included the effect of novelty on utility. The novelty of an option ($N({s}_{i})$) was defined as the normalized variance of a beta distribution specified according to the number of times a particular stimulus had been observed across the entire trials (beta [$\alpha =$ number of exposures + 1; $\beta =$ 1]), as in the logistic regression analysis. As in the previous study, we originally planned to include novelty bias by inflating the initial values of the hyperparameters of the Beta distribution (${\alpha }_{0}$ and ${\beta }_{0}$) of each option (i.e., optimistic/pessimistic initialization) such that:
  8 $$\alpha_0=1+B\;\mathrm{when}\;B>0$$
  9 $$\beta_0=1+\left|B\right|\mathrm{when}\;B<0$$
  where *B* is a free parameter that determines the degree of novelty bias. However, we could not find a good gradient for several subjects when we conducted the first-level fitting with this model (see the “Parameter Estimation and Model Comparison” section for a detailed procedure). This was considered to be due to the fairly high estimated values of the forgetting rate in those participants (see Fig. 6a). When the forgetting rate is high, novelty initialization bias quickly disappears and parameter estimation for the bias is indicated to become unstable (Cockburn et al., 2022). In this study, therefore, we represented novelty bias as a bonus feature included in utility. This means that the agent calculated utility by adding weighted novelty to the *q*-value:
  10 $$V\left({s}_{i}\right)=Q\left({s}_{i}\right)+{w}_{N}\cdot{N}\left({s}_{i}\right)$$
  This representation was also considered in the previous study and was found to be behaviorally indistinguishable from the optimistic initialization method (Cockburn et al., 2022). This model allowed for the successful estimation of parameters. Free parameters of this model were $\eta$, $\beta$, and ${w}_{N}$.
3. Uncertainty bias model (four parameters): The uncertainty bias model included the effect of uncertainty on utility. Specifically, weighted uncertainty of an option ($U({s}_{i})$), which was defined as a normalized variance of the Beta distribution specified according to the number of wins and losses within a block, was added to utility:
  11 $$V\left({s}_{i}\right)=Q\left({s}_{i}\right)+{w}_{t}^{U}\cdot{U}\left({s}_{i}\right)$$
  The weight (${w}_{t}^{U}$) was assumed to change linearly depending on the task horizon, as in Cockburn et al. (2022):
  12 $${w}_{t}^{U}={U}_{I}+ \frac{\left(t-1\right)\cdot\left({U}_{T}-{U}_{I}\right)}{{T}_{block}}$$
  where ${U}_{I}$ and ${U}_{T}$ are parameters that denote the initial and terminal value of the weight respectively, and ${T}_{block}$ is the maximum number of trials in the block (i.e., 20). Parameters of this model were $\eta$, $\beta$, ${U}_{I}$, and ${U}_{T}$.
4. Novelty and uncertainty bias model (five parameters): This model included the effects of both novelty and uncertainty on utility:
  13 $$V\left({s}_{i}\right)=Q\left({s}_{i}\right)+{w}_{N}\cdot{N}\left({s}_{i}\right)+{w}_{t}^{U}\cdot{U}\left({s}_{i}\right)$$
  Parameters were $\eta$, $\beta$, ${w}_{N}$, ${U}_{I}$, and ${U}_{T}$.
5. Familiarity-gated uncertainty model (four parameters): In this model, uncertainty bias was modulated by familiarity of an option. Familiarity ($F\left({s}_{i}\right)$) was calculated based on novelty:
  14 $$F\left({s}_{i}\right)=1-N\left({s}_{i}\right)$$
  When calculating utility, familiarity modulated the effect of uncertainty such that uncertainty bias was suppressed when presented stimulus was novel:
  15 $$V\left({s}_{i}\right)=Q\left({s}_{i}\right)+F\left({s}_{i}\right)\cdot{w}_{t}^{U}\cdot{U}\left({s}_{i}\right)$$
  Parameters were $\eta$, $\beta$, ${U}_{I}$, and ${U}_{T}$.
6. Novelty-biased Familiarity-gated uncertainty model (five parameters): This model included both novelty bias and familiarity-modulated uncertainty bias when calculating utility:
  16 $$V\left({s}_{i}\right)=Q\left({s}_{i}\right)+{w}_{N}\cdot{N}\left({s}_{i}\right)+F\left({s}_{i}\right)\cdot{w}_{t}^{U}\cdot{U}\left({s}_{i}\right)$$
  Parameters were $\eta$, $\beta$, ${w}_{N}$, ${U}_{I}$, and ${U}_{T}$.

To allow for between-condition difference in the behavior at the group-level, we also modeled a difference parameter (${D}_{param}$) for each free parameter of interest. Specifically, the free parameters in the high reward and low reward conditions were represented as follows, respectively:

17 $${Param}_{high\;reward}=Param+\frac{{D}_{param}}{2}$$

18 $${Param}_{low\;reward}=Param-\frac{{D}_{param}}{2}$$

Note that this way of representing the between-condition differences in the parameters is not what was preregistered. Originally, we planned to perform model fitting separately for each condition and then compare the estimated parameters using statistical tests. However, based on feedback received during the peer review process, which highlighted an increased risk of false positives when applying conventional statistical tests to hierarchically fitted data (Boehm et al., 2018), we revised our approach to express the differences within the model. The main results were consistent between the original and revised versions. See version 1 of the preprint (https://doi.org/10.21203/rs.3.rs-4627464/v1) for the original modeling methods and results.

#### Parameter Estimation and Model Comparison

Parameter estimation and model comparison were conducted using Hierarchical Bayesian inference (HBI; Piray et al., 2019), with the computational and behavioral modeling toolbox of Matlab R2021b (The MathWorks Inc, 2020). The HBI framework enables concurrent model comparison and hierarchical estimation of parameters. This approach assumes random effects for model comparison, which makes it possible to estimate parameters while taking into account the possibility that the model used in the population differs between individuals.

We followed the procedure of the previous studies (Cockburn et al., 2022; Nussenbaum et al., 2023). First, each candidate model, including the difference parameters, was fit to the choice data of each participant using common priors ($N\left(\mu =0, {\sigma }^{2}=6.25\right)$) in a non-hierarchical way. Forgetting rate ($\eta$) and inverse temperature ($\beta$) were transformed to have a range of 0–1 and 0–20, respectively. These first-level estimates were then used to form empirical priors in the second-level, hierarchical fitting procedure and concurrent model comparison. Specifically, the second fitting procedure included the following 4 steps: (1) calculating the summary statistics, (2) updating the group-level posterior of parameters, (3) updating the individual-level posterior of parameters, and (4) updating the estimated responsibility of each model in generating each individual’s data. In this way, we obtained the group-level and individual-level estimates of parameters under the assumption that participants might have used different models (see Piray et al. (2019) for mathematical details). The best model was selected based on protected exceedance probability (PXP), which is the probability that a given model is more frequent than all other candidate models in the population, taking into account the possibility that any difference between model frequencies is due to chance (Rigoux et al., 2014). See Supplementary Information for results regarding parameter recovery and model recovery (Fig. S1 and S2).

#### Analysis with Estimated Parameters

Whether the estimated group-level parameters of the Bayesian RL models significantly differed from zero was tested using the HBI *t*-test (Piray et al., 2019). In the HBI *t*-test, it is possible to determine whether any group-level parameter of a given model significantly differs from an arbitrary value by using the fact that the parameter follows a Student’s *t*-distribution centered on the group mean. Unlike the classical Student’s *t*-test, the degrees of freedom of the distribution are determined by the estimated number of subjects whose behavior is best explained by the corresponding model, not by the total number of subjects. When a parameter value in the HBI *t*-test is found to differ from zero with *p* < 0.05, it means that the 95% credible interval of the posterior distribution for that group-level parameter does not include 0 (see Piray et al. (2019) for mathematical details).

We tested whether the estimated group-level parameters of the best model significantly differed between conditions by conducting the HBI *t*-test on the difference parameters (${D}_{param}$) of the corresponding parameters. That is, if ${D}_{param}$ significantly differed from zero with *p* < 0.05, the corresponding parameter was considered to be significantly different between conditions.

##### Preregistered Expected Results

In the first logistic regression model (Eq. 1), we expected that, as in the previous studies (Cockburn et al., 2022; Nussenbaum et al., 2023), the difference in expected win probability (${W}_{\Delta }$) and novelty (${N}_{\Delta }$) would show significant positive effects on choice, while the difference in uncertainty (${U}_{\Delta }$) would have a significant negative effect on choice, reflecting reward- and novelty-directed choices and uncertainty-aversive choices. Regarding the behavioral differences between high reward and low reward conditions, we expected that there would be a significant positive interaction between the level of reward expectancy and the difference in expected reward probability (${W}_{\Delta }*M$), reflecting an increase in sensitivity to the difference in reward likelihood and possibly a decrease in random exploration when relative reward expectancy was high. We expected that an interaction between the level of reward expectancy and the difference in uncertainty (${U}_{\Delta }*M$) would also be significantly positive, reflecting an increase in uncertainty-directed exploration.

As for Bayesian RL modeling, we expected that the “novelty-biased familiarity-gated uncertainty model” would be selected as the best model, based on the results of adults in the previous studies (Cockburn et al., 2022; Nussenbaum et al., 2023). We did not preregister specific hypotheses about the estimated parameters since we did not know what model would be selected as the best model. However, the inverse temperature ($\beta$) was expected to be higher in the high reward condition, reflecting a decrease in random exploration, while the uncertainty bias parameters (${U}_{I}$ and ${U}_{T}$) were expected to be higher in the high reward condition, reflecting an increase in uncertainty-directed exploration. In other words, we expected that the difference parameters of these (${D}_{\beta }$, ${D}_{{U}_{I}}$, and ${D}_{{U}_{T}}$) would be positive and significantly different from zero.

## Results

### Difference in Task Performance

**Fig. 2:**
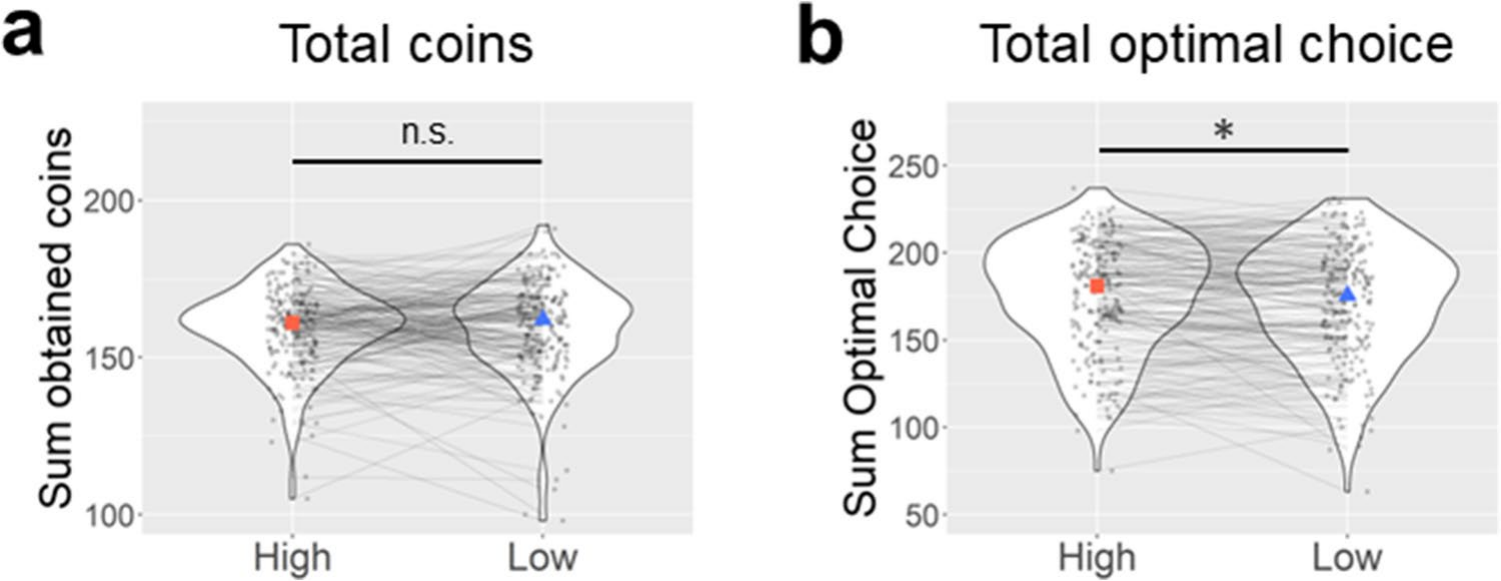
Participants’ performance in the two-armed bandit task in high and low reward conditions. **a** Total number of obtained coins in the two conditions. Each point corresponds to each participant, and red rectangle and blue triangle represent the median of all participants. **b** Total number of optimal choices in the two conditions. Each point corresponds to each participant, and red rectangle and blue triangle represent the median of all participants

Participants did not differ in their total number of obtained coins between high and low reward conditions (median of high reward condition: 161, median of low reward condition: 162, *t*(197) = 0.01, 95% CI = [$-$ 2.03, 2.10], *p* = 0.99, Hedges’ *g* = 0.00, Fig. 2a). However, participants made slightly but significantly more frequent optimal choices in the high reward condition compared with the low reward condition (median of high reward condition, 181; median of low reward condition, 176; median difference = 3.50, *p* = 0.006, 95% CI = [1.00, 6.00], Fig. 2b). This indicates that, in general, participants behaved in a more optimal way in the high reward condition compared with the low reward condition.

### The Effect of Interaction Between Reward Magnitude and Reward Probability, Uncertainty, and Novelty on Choice

**Fig. 3:**
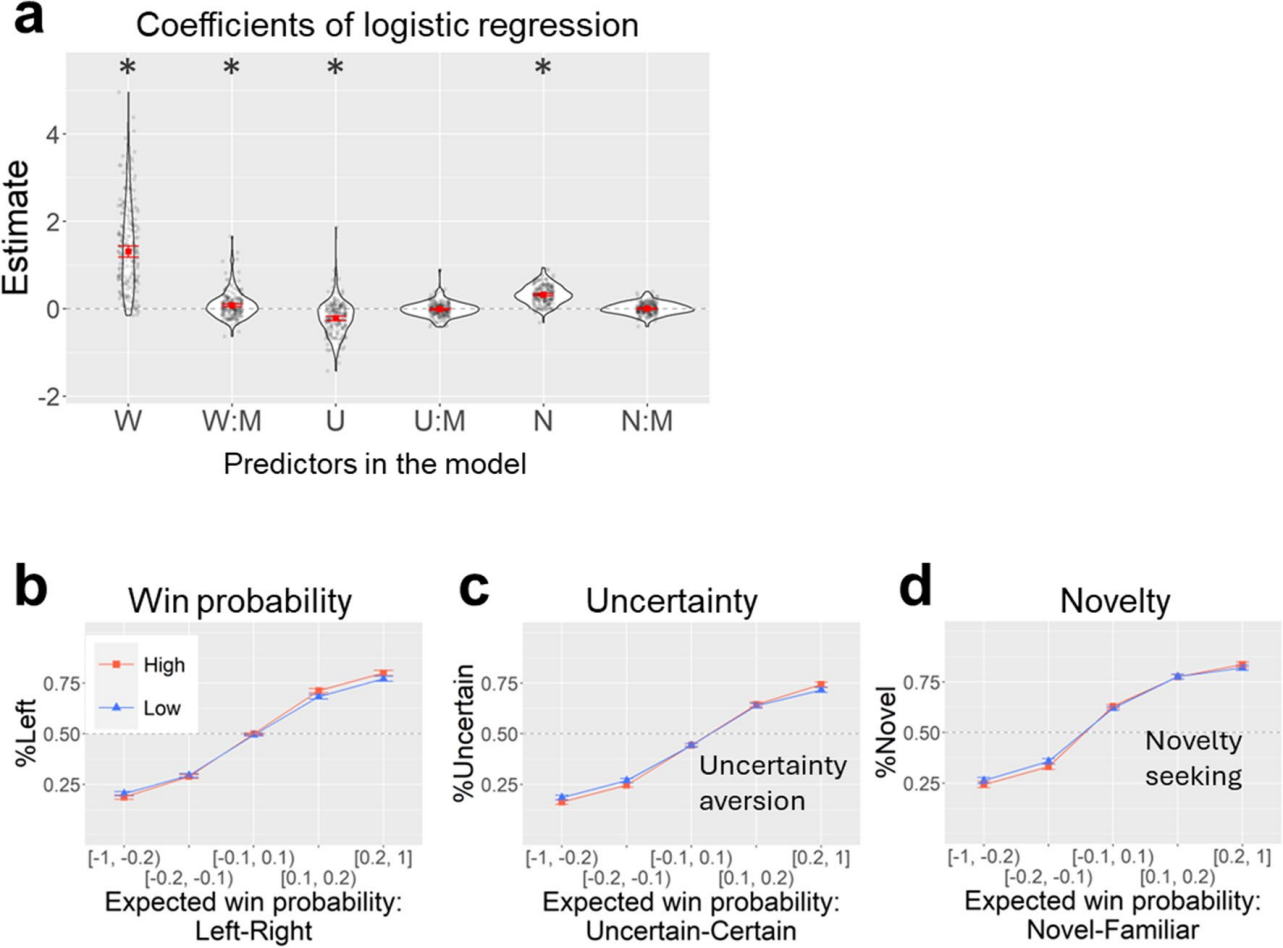
Visualization of the results of logistic regression. **a** The group-level estimates of coefficients (red points) and random effects (gray dots) from logistic regression examining the effects of expected win probability (W), uncertainty (U), novelty (N), and their interaction with reward magnitude (M) on choice. The asterisks at the top indicate statistically significant predictors. **b** Proportion of choosing the left stimulus as a function of the difference in expected win probability. The horizontal axis represents various ranges of the difference in expected win probability (e.g., [0.2, 1] means that the difference lies between 0.2 and 1. The same applies to **c** and **d**). The red rectangle and blue triangle correspond to high and low reward conditions, respectively (the same applies to **c** and **d**). **c** Proportion of choosing the more uncertain stimulus, when two familiar stimuli (observed more than three times) were presented, as a function of the difference in expected win probability. **d** Proportion of choosing the more novel stimulus, when one of the two stimuli was novel (observed less than three times), as a function of the difference in expected win probability

To examine the difference in the effects of reward probability, uncertainty, and novelty on the choice between high and low reward conditions, we conducted a mixed-effects logistic regression analysis. The results are summarized in Fig. 3a. Participants showed a general preference for options with higher reward probability (${\beta }_{{W}_{\Delta }}$ = 1.31, *p* < 2 $\times$ 10^−16^) and novelty (${\beta }_{{N}_{\Delta }}$ = 0.32, *p* < 2 $\times$ 10^−16^), while they avoided more uncertain options (${\beta }_{{U}_{\Delta }}$ = $-$ 0.22, *p* < 2 $\times$ 10^−16^), in line with the results of a previous study (Cockburn et al., 2022). Reward magnitude showed a significant positive interaction with reward probability (${\beta }_{{M:W}_{\Delta }}$ = 0.08, *p* = 2.3 $\times$ 10^−7^), which indicates that participants were more sensitive to the difference in reward probability upon choice, as can be seen in the slope of Fig. 3b. The interaction between reward magnitude and uncertainty (${\beta }_{{M:U}_{\Delta }}$ = $-$ 0.01, *p* = 0.32) and the interaction between reward magnitude and novelty (${\beta }_{{M:N}_{\Delta }}$ = 0.00, *p* = 0.61) had no significant impacts on choice, indicating that there was no difference in novelty- and uncertainty-based exploration in high and low reward conditions (Fig. 3c and d).

**Fig. 4:**
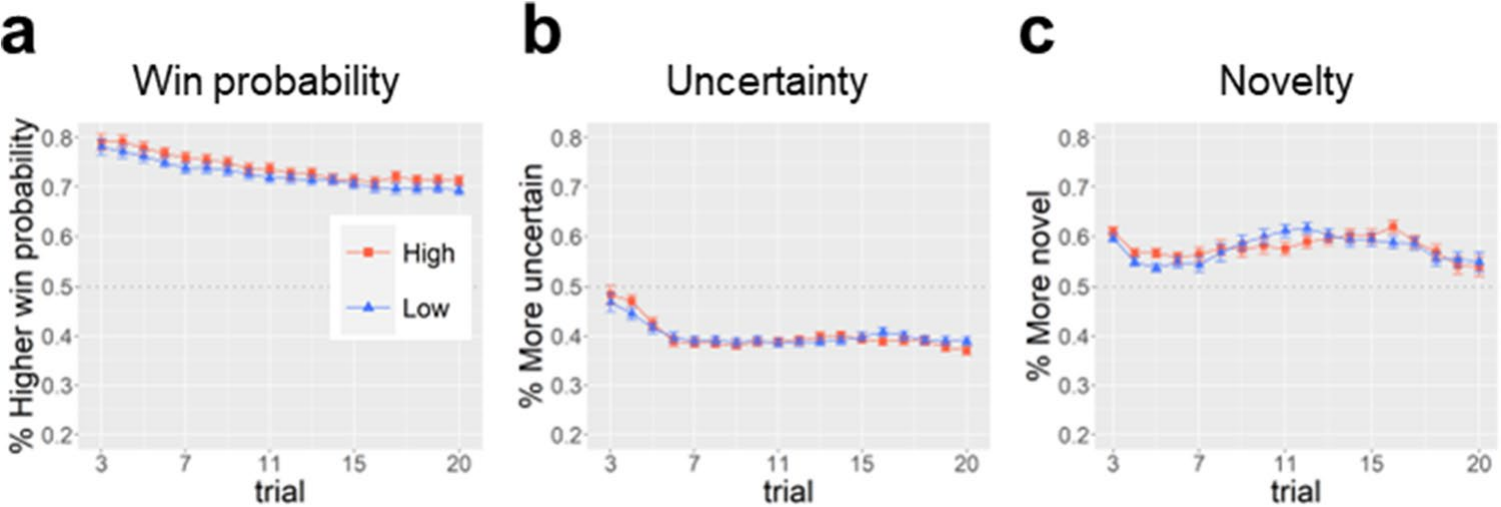
The effect of the interaction between task horizon and expected win probability, uncertainty, and novelty on choice in each condition. The red rectangle and blue triangle correspond to high and low reward conditions, respectively. **a** Within-blocks temporal change in the moving average of the proportion of choosing the option with higher expected win probability, averaged across blocks and subjects. The time window for the moving average was three trials (the same applies to **b** and **c**). **b** Within-blocks temporal change in the moving average of the proportion of choosing the option with higher uncertainty, averaged across blocks and subjects. **c** Within-blocks temporal change in the moving average of the proportion of choosing the option with higher novelty, averaged across blocks and subjects

Next, as an exploratory analysis, we sought to examine if there were differences between high and low reward conditions in the effect of task horizon on choice. Participants’ preference for options with higher expected reward probability generally decreased (${\beta }_{{t:W}_{\Delta }}=-0.60, p<2\times {10}^{-16}$, Fig. 4a), which was considered to be due to the forgetting effect (see Supplementary Information). The proportion of choosing more uncertain options generally decreased (${\beta }_{{t:W}_{\Delta }}=-0.08, p=3.5\times {10}^{-4}$, Fig. 4b) as the block approached its end. It appears that the preference for novelty is maintained at a relatively constant level despite fluctuations, although the coefficient was negative (${\beta }_{{t:N}_{\Delta }}=-0.04, p=.02$; Fig. 4c). Such interactions with task horizon, however, did not show any significant interaction with reward magnitude (${\beta }_{{M:t:W}_{\Delta }}=0.01, p=.71;\;{\beta }_{{M:t:U}_{\Delta }}=-0.04, p=.12;\;{\beta }_{{M:t:N}_{\Delta }}=-0.01, p=.60$, Fig. 4c). This indicates that temporal changes in the effect of reward probability, uncertainty, and novelty on choice were equivalent in the two conditions, as can be seen in Fig. 4a–c.

### The Difference in the Estimated Parameters of the Bayesian Reinforcement Learning Model Between the High and Low Reward Conditions

The logistic regression analysis showed that the more optimal choices in the high reward condition were mainly due to increased sensitivity to reward probability, and that there was no difference in the influence of uncertainty and novelty on choice between the two conditions. Several possibilities could explain the mechanism behind the increased sensitivity to the difference in reward probability. For instance, it could be due to a decrease in the forgetting rate, allowing outcomes to be more accurately reflected in value, or a reduction in random exploration leading to greedier decision-making, or perhaps a combination of both. We fitted choice data in the two conditions with six variants of forgetful Bayesian RL models to gain insight into the detailed mechanisms behind the behavioral differences between the two conditions. In these models, the agent represents each option with a Beta distribution and updates parameters of the distribution based on observations of outcomes (i.e., whether a coin was obtained or not), assuming that it gradually forgets past observations (see the “Bayesian Reinforcement Learning Modeling” section for details and see Supplementary Information for the rationale for including the forgetting effect in our models).

As a result of Hierarchical Bayesian inference (HBI; Piray et al., 2019), the “novelty-biased familiarity-gated uncertainty model” was selected as the best model (PXP = 0.70), consistent with the previous study (Cockburn et al., 2022). In line with the results of the logistic regression analysis, the group-level initial and terminal uncertainty bias parameters both showed significantly negative values (${{U}_{I}}_{group\;mean}$ = $-$ 0.14, *t*(83.71) = $-$ 15.18, *p* = 9.08 $\times$ 10^−26^; ${{U}_{T}}_{group\;mean}$ = $-$ 0.34, *t*(83.71) = $-$ 30.28, *p* = 7.18 $\times$ 10^−47^), reflecting the uncertainty-aversive behavior of participants. Unexpectedly, the group-level novelty bias parameter did not show a significantly positive value (${{w}_{N}}_{group\;mean}$ = 0.01, *t*(83.71) = 0.81, *p* = 0.42), although the logistic regression analysis indicated a preference for novel options. In the “novelty-biased familiarity-gated uncertainty model,” the influence of uncertainty on utility is suppressed by the novelty of stimuli (Eq. 16). This means that when uncertainty bias is negative, the relative values of novel options tend to be higher compared to familiar options, because novel options are unaffected by negative uncertainty bias. In other words, the preference for novel stimuli can be represented solely by the modulating effect of familiarity on uncertainty (Nussenbaum et al., 2023). The observed preference for novelty in our study could be sufficiently explained by the modulation effect alone, which might be why ${w}_{N}$ was not significantly positive. In fact, it was possible to reproduce participants’ behavior in simulations using the estimated parameters (see Supplementary Information).

**Fig. 5:**
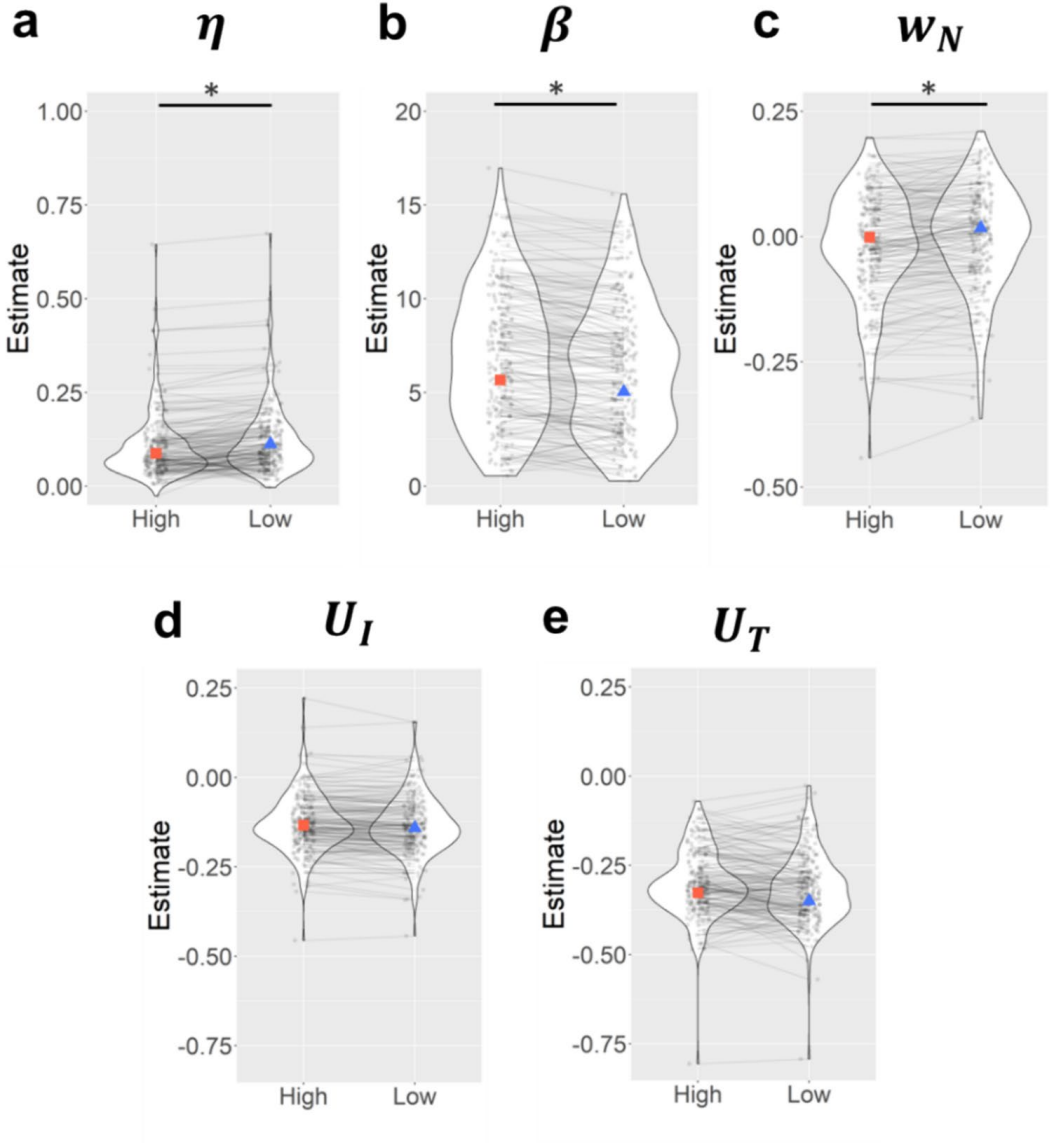
The estimated parameters of the “novelty-biased familiarity-gated uncertainty model.” Red rectangles and blue triangles represent the group-level estimates of each parameter in the high and low reward conditions, respectively, and each gray point represents the individual-level estimates. Each parameter value was calculated by using the difference parameter (i.e., $Param\pm {D}_{param}/2$). Asterisks indicate that the difference parameter for that parameter was significantly different from zero. **a** Forgetting rate. **b** Inverse temperature. **c** The novelty bias parameter. **d** The initial uncertainty bias parameter. **e** The terminal uncertainty bias parameter

To examine whether the parameters of the best model differed between conditions, we modeled within-participant difference parameters (${D}_{param}={Param}_{high\;reward}-{Param}_{low\;reward}$) for each free parameter and tested whether the group-level ${D}_{param}$ significantly differed from zero using the HBI *t*-test (see the “Methods” section for details). The forgetting rate was found to be significantly lower in the high reward condition (${\eta }_{group\;mean}$ = 0.10, ${D}_{\eta }$ = $-$ 0.02, *t*(83.71) = $-$ 3.83, *p* = 2.45 $\times$ 10^−4^, Fig. 5a), indicating that participants reflected past outcomes on utility with less decay when reward magnitude was high. The inverse temperature was estimated to be significantly higher in the high reward condition (${\beta }_{group\;mean}$ = 5.34, ${D}_{\beta }$ = 0.63, *t*(83.71) = 4.10, *p* = 9.39 $\times$ 10^−5^, Fig. 5b), suggesting that participants engaged in less random exploration (i.e., showed less noise in decision-making) when reward magnitude was high. In addition, the novelty bias parameter was significantly lower in high reward condition (${{w}_{N}}_{group\;mean}$ = 0.01, ${D}_{{w}_{N}}$ = $-$ 0.02, *t*(83.71) = $-$ 2.57, *p* = 0.01, Fig. 5c), indicating that the influence of novelty, relative to that of reward probability, on utility was weaker when reward magnitude was high. On the other hand, the initial uncertainty bias and terminal uncertainty bias parameters did not show significant differences between conditions (${{U}_{I}}_{group\;mean}$ = $-$ 0.14, ${D}_{{U}_{I}}$ = 0.01, *t*(83.71) = 1.06, *p* = 0.29, Fig. 5d; ${{U}_{T}}_{group\;mean}$ = $-$ 0.34, ${D}_{{U}_{T}}$ = 0.02, *t*(83.71) = 1.68, *p* = 0.10, Fig. 5e).

## Discussion

In this study, we examined the influence of environmental richness on random and directed exploration by manipulating the reward magnitude in a two-armed bandit task designed to dissociate uncertainty and novelty. Participants showed increased sensitivity to differences in reward probability and made more frequent optimal choices when relative reward magnitude was high, but there appeared to be little effect of reward magnitude on the degree of uncertainty- and novelty-based exploration, according to the results of logistic regression analysis. Bayesian RL modeling revealed that when the relative reward magnitude was high, participants exhibited a lower forgetting rate and a higher inverse temperature. These findings suggest that participants more accurately incorporated past outcomes when representing the utility of options and engaged in less random exploration. In line with the results of logistic regression analysis, there were no significant differences in initial and terminal uncertainty bias between the conditions, although the novelty bias was smaller in the high reward condition (see below for a discussion on novelty-based exploration). We therefore conclude that, based on both logistic regression and Bayesian RL modeling results, while random exploration decreased with higher reward magnitude, the degree of uncertainty-based directed exploration remained largely unchanged. In other words, our first hypothesis was supported while our second hypothesis was not.

Regarding novelty-based exploration, the results from logistic regression and computational modeling seem to contradict each other. Although the novelty bias parameter $\left({w}_{N}\right)$ significantly differed between high and low reward conditions, the preference for novelty and its temporal trajectory did not show significant differences between the two conditions. This is considered to be because in the Bayesian RL model, the eventual effects of novelty (and uncertainty) on choice are determined by the products of the inverse temperature and the bias parameter ($\beta *{w}_{N}*N\left({s}_{i}\right)$; see Eqs. (7) and (16)). This means that the degree of novelty-based exploration depends not only on the bias parameters but also on the inverse temperature. It is therefore possible that the simultaneous increase in inverse temperature resulted in an almost unchanged effect of novelty on behavior.

Bayesian RL modeling revealed that the increase in optimal choices in the high reward condition was due to both less forgetting and less random exploration. The result that participants showed less forgetting in the high reward condition is in line with previous research showing that reward magnitude enhances goal-directed or proactive behavior (Chiew & Braver, 2011; Rosell-Negre et al., 2017). Participants are considered to have been more motivated in the high reward condition than in the low reward condition, as their choices in the high reward condition offered greater practical benefits, with no monetary costs in either condition. Such incentives have been shown to facilitate the use of a model-based system during reinforcement learning tasks, which is thought to be supported by executive functions and imposes a greater cognitive load than the model-free system (Bolenz & Eppinger, 2022; Kool et al., 2017). Thus, a higher reward magnitude might have promoted executive function, including working memory, leading to less forgetting of observations in the high reward condition.

Consistent with our hypothesis, participants showed less random exploration with higher reward magnitude, indicated by a larger estimated value of inverse temperature in high reward condition. It has been both theoretically suggested and empirically demonstrated that pharmacological dopaminergic inhibition can lead to increased random choices without affecting learning rates, by reducing of the amplitude of reward prediction error (RPE; Cinotti et al., 2019). Specifically, Cinotti et al. (2019) mathematically showed that when positive RPEs were scaled by a factor *g*, the learned *Q*-values were also scaled by *g*, and that the softmax function using the scaled *Q*-values was equivalent to the softmax function using the original (unscaled) *Q*-values and a scaled inverse temperature multiplied by *g*. They then experimentally showed that, using rats, dopaminergic inhibition could actually increase random choices (i.e., decrease in inverse temperature). Based on this finding, one possible explanation for the reduced random exploration observed in our study is the change in the magnitude of RPE. It is conceivable that the increased magnitude of reward in the high reward condition strengthened the overall level of RPEs and the accompanying phasic dopamine activity, resulting in less decision noise within the condition.

In contrast to the results regarding random exploration, we observed little effect of reward magnitude on uncertainty-directed exploration. Humans are shown to engage in more uncertainty-based directed exploration when the task horizon is longer in order to gain information that can help future decisions (Cockburn et al., 2022; Wilson et al., 2014). We therefore expected that high reward magnitude would drive information gathering and thus lead to more uncertainty-based exploration. However, while we observed a dependency of the effect of uncertainty on task horizon similar to the previous study (Cockburn et al., 2022), the change in reward magnitude affected neither the overall influence of uncertainty on behavior nor its temporal trajectory. This suggests that the degree of directed exploration in human reinforcement learning might not depend on the magnitude of reward prediction in the current environment. One possibility, however, should be noted that the lack of difference could be attributed to the design of the two-armed bandit task in this study. The longest task horizon in this study (i.e., the number of trials per block) was 20, which is relatively short compared to a non-stationary four-armed bandit task commonly used to study directed exploration (Daw et al., 2006). Due to this limited length and stationary design, simply increasing directed exploration might not have led to obtaining more reward. In fact, our supplementary simulation showed that the total number of optimal actions had an inverted U-shaped relationship with the initial weight parameter for uncertainty (${U}_{I}$), taking the maximal value when the initial uncertainty bias was close to zero (see Fig. S6). According to this result, participants in our study exhibited nearly optimal initial uncertainty bias (i.e., near zero) in both low and high reward conditions. It is therefore possible that participants showed no change in the degree of uncertainty-based exploration depending on reward expectancy because there was no better strategy for uncertainty-based behavior (i.e., because of the ceiling effect).

While such an explanation can be considered, the results of our study suggest that reward magnitude decreases random exploration but has little impact on directed exploration. This implies that, in general, humans might behave in a more exploitative way when the environment is relatively rich. This seemingly contradicts previous findings suggesting that high reward frequency contingent on actions enhances exploration for unknown options in humans (Teodorescu & Erev, 2014) and decreases exploitative behavior through modulation of the balance of positive and negative learning rates in rats (Ohta et al., 2021). However, as stated in the “Introduction” section, the critical aspect of our study is that we manipulated environmental richness independently of controllability, allowing us to examine the pure influence of the richness alone on exploration. It is conceivable, therefore, that the increased exploration observed in previous studies could be attributed to changes in controllability (which was caused by manipulation of reward probability) rather than the level of environmental richness. Given that controllability is thought to be a core component of learned helplessness (Maier & Seligman, 2016), and that an agent experiencing learned helplessness exhibits a lack of explorative behavior in new environments, it is reasonable to expect that controllability also has a causal impact on random and/or directed exploration. The role of controllability in human reinforcement learning has recently been investigated in some studies (Dorfman & Gershman, 2019; Gershman et al., 2021), but its relationship with different types of exploration has not been directly tested. Future studies should examine the influence of controllability on random exploration and uncertainty- and novelty-based directed exploration, although it is challenging to manipulate controllability alone without confounding with reward expectations contingent on actions.

Our study has several limitations. One limitation is related to the task design. In our two-armed bandit task, participants proceeded immediately to the next trial if the first outcome was no reward (i.e., no coin). This setting, combined with relatively short task horizons within blocks, might have compromised the learning of participants. For example, if a coin was not obtained by chance when selecting the stimulus with the highest true reward probability within the block, participants might have ended the block without subsequently selecting that stimulus again. This could lead to objectively suboptimal learning and decision-making. This issue could be addressed by extending the task horizon within blocks, which might also alleviate the potential ceiling effect of uncertainty-based exploration mentioned earlier. Another limitation related to the immediate transition to the next trial upon losing is that losing in the low reward condition might have increased the eventual size of the bonus reward per unit time, potentially motivating participants to behave less optimally in the low reward condition. Our post-hoc analysis, however, indicated that participants remembered the stimuli used in the two conditions equally well (${b}_{condition}=-0.002, p= .86$; see Fig. S4). This might be one evidence suggesting that participants were motivated to maximize rewards in both conditions. A third limitation is that the sample consisted only of young adults. Given that exploration strategies can vary with age (Liquin & Gopnik, 2022; Nussenbaum et al., 2023), the effects of reward magnitude observed in this study might differ across developmental stages.

To conclude, this study found that higher reward magnitude reduces random exploration but has little impact on uncertainty- and novelty-based directed exploration. It offers evidence that humans flexibly modulate the balance between exploration and exploitation depending on environmental richness. Given that general reward expectations fluctuate within individuals depending on daily life experiences (Shimomura et al., 2023), it is likely that the degree of exploration shows state-dependent intraindividual changes, as indicated by a recent study reporting within-individual temporal variability in reinforcement learning parameters, including inverse temperature (Schaaf et al., 2023). It is an important direction to examine aspects that can cause a dynamic change in the exploration–exploitation balance for understanding how humans resolve this dilemma in complex real-world environments.

## Supplementary Information

Below is the link to the electronic supplementary material.

Supplementary file1 (DOCX 788 KB)

## Data Availability

This study is preregistered. In addition to the preregistered document, all the data and codes that support the findings of this study is available on https://osf.io/tjba9/.
