# Supplementary material for: The Effect of Reward Magnitude on Different Types of Exploration in Human Reinforcement Learning
Source: Comput Brain Behav. 2024 Oct 3;8(1):147–61. doi: 10.1007/s42113-024-00224-6 (PMC13298669; doi:10.1007/s42113-024-00224-6)
Supplement: Supplementary file 1 — Supplementary file1 (DOCX 788 KB) [file 42113_2024_224_MOESM1_ESM.docx]

**Supplementary information for “The effect of reward magnitude on different types of exploration in human reinforcement learning”**

**Journal name**

*Computational Brain and Behavior*

**Authors & Affiliations**

Kanji Shimomura^1,2^* & Kenji Morita^1,3^

1 Graduate School of Education, The University of Tokyo, Tokyo, Japan

2 Japan Society for the Promotion of Science, Tokyo, Japan

3 International Research Center for Neurointelligence (WPI-IRCN), The University of Tokyo, Japan

*Corresponding author

Kanji Shimomura (skcpccp@gmail.com, ORCID: 0000-0003-4370-3710)

***Parameter recovery***

We conducted parameter identifiability analysis with the best model in this study (i.e., “novelty-biased familiarity-gated uncertainty model”). Specifically, first, we pseudo-randomly generated 150 parameter sets using uniform distribution and generated 100 data (each consisting of 300 trials) with each parameter set. The ranges (lower and upper bounds) of uniform distribution for generating each parameter were as follows: $\eta$: 0 to 1, $\beta$: 0 to 20, $N$: $-$2 to 2, $W_{I}$: $-$2 to 2, and $W_{T}$: $-$2 to 2. We then conducted model fitting in the same way as stated in the main text using the generated datasets and estimated parameters for each parameter set.

The results of parameter recovery are summarized in Fig. S1. As shown in Fig. S1, all five parameters showed high positive correlations ($\eta$: 0.98, $\beta$: 0.92, $w_{N}$: 0.96, $U_{I}$: 0.96, and $U_{T}$: 0.96), indicating that parameter identifiability of the model was sufficiently high.


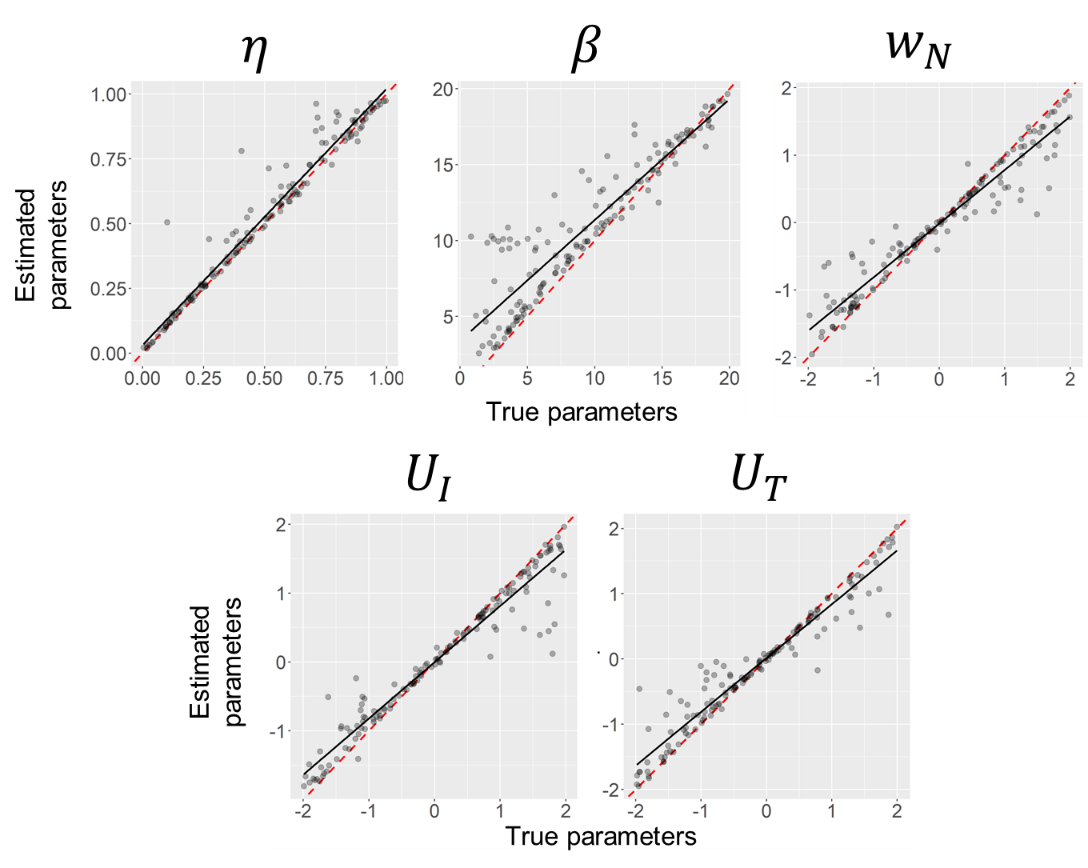


**Fig. S1** Correlation between true (generative) parameters and estimated parameters of novelty-biased familiarity-gated uncertainty bias model. Horizontal axis represents true values of each parameter and vertical axis shows estimated values of each parameter. Red dashed line represents identity line (i.e., y=x), and black line represents best fit linear regression line.

***Model recovery***

To examine if the candidate models in our study were identifiable, we generated 50 simulated datasets with each model using participants’ trial sequence and random parameter sets derived from uniform distributions. The range of the uniform distributions were determined by the minimum and maximum values of individual estimated parameters. We then conducted model fitting procedures reported in the main text using the generated datasets and examined model frequencies and protected exceedance probabilities (PXPs) for each model.

Fig. S2 shows the results of model recovery. With the parameter ranges observed in the present study, all models except for “familiarity-gated uncertainty model” were sufficiently recoverable. For data generated by the "familiarity-gated uncertainty bias model," the "novelty-biased familiarity-gated uncertainty model" was almost always selected as the best model. The only difference between the "familiarity-gated uncertainty model" and the "novelty-biased familiarity-gated uncertainty model" is the presence of the novelty bias parameter. If the value of the novelty bias parameter is zero, the latter becomes equivalent to the former. Additionally, as mentioned in the text, even if the novelty bias parameter is zero (that is, even if we use the “familiarity-gated uncertainty bias model”), it is possible to represent a preference for novelty through the effect of novelty suppressing the influence of uncertainty. These factors are likely to make it difficult to distinguish between the two models. In fact, similar results were also reported by Nussenbaum et al. (2023).

In the present study, the "novelty-biased familiarity-gated uncertainty model" was selected as the best model. However, the estimated group mean of the novelty bias parameter was very small (${w_{N}}_{group mean}$ = 0.01) and was not significantly different from zero (see the main text), making it almost equivalent to the "familiarity-gated uncertainty model." Therefore, even if the "familiarity-gated uncertainty model" was the true model in this study, we conceive that it would not affect the conclusions.


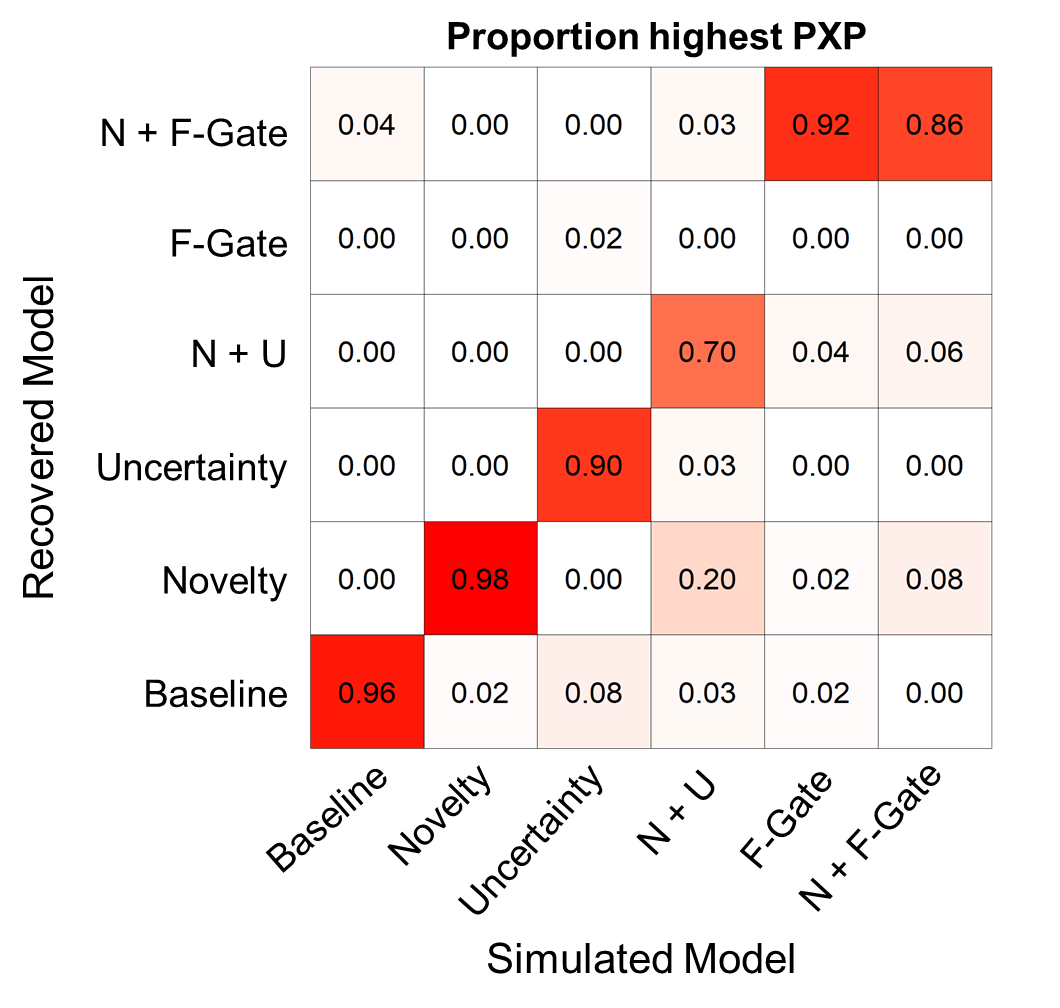


**Fig. S2** Confusion matrices showing the probability of each fitted model given a simulated model. Specifically, it represents the proportion of the 50 fittings performed on data generated by a simulated model in which a given model had the highest protected exceedance probability (PXP). Baseline: Baseline model, Novelty: Novelty bias model, Uncertainty: Uncertainty bias model, N + U: Novelty and uncertainty bias model, F-Gate: Familiarity-gated uncertainty bias model, N + F-Gate: Novelty-biased familiarity-gated uncertainty model.

***Uncertainty manipulation check***

In the two-armed bandit task adopted in this study, the reward probabilities of options were reset at each block to dissociate uncertainty from novelty. We explicitly told participants about this structure (for example, we instructed as follows: “Importantly, even for the same painting, the probability of earning a coin is independent across casinos (blocks)”), and they could start the experiment only after they correctly answered to the questions regarding the task, which included the item about the abovementioned block structure. However, it is still possible that they did not fully understand this structure or even if they understood, learned values in one block might have automatically propagated to the subsequent blocks.

To confirm that participants really understood the structure and reset values at each block, we conducted model comparison between the “novelty-biased familiarity-gated uncertainty model” with value reset and without value reset between blocks. Specifically, we fitted the two models to participants’ choice data and tested which model is selected as the best model based on PXP as was done in the main text. As a result, the model with value reset was strongly favored (PXP = 1.00, model frequency=0.94). This confirmed that participants clearly understood the task structure and did not carry over the values learned in previous blocks to subsequent ones.

***Possible effects of the order of the conditions***

We observed increased sensitivity to reward in high reward condition, and this was explained by increased inverse temperature (combined with decreased forgetting rate) in the condition. It is possible that these differences were at least partially accounted for by the effect of the order of conditions that participants experienced. To examine the effect of the order of conditions, we conducted a logistic regression with a new model including the order of conditions (“$O$”) as an additional independent variable ($p\left( A_{t}=L \right)=\left( W_{\Delta}+U_{\Delta}+N_{\Delta} \right)*M*O+\left( 1 | ID \right)$). The order of conditions was coded as 0 if the high reward condition was experienced first and 1 if the low reward condition was experienced first. In addition, we examined whether the mean estimated difference parameter for inverse temperature ($D_{\beta}$) significantly differed between participants experiencing the high reward condition first and participants experiencing the low reward condition first, using a t-test.

The logistic regression analysis revealed some effects of the order of conditions. Specifically, there was a significant positive interaction between the order and reward probability ($\beta_{{O:W}_{\Delta}}=0.03$, *p* = 1.3$\times$10^-5^), indicating that participants who experienced the low reward condition first were generally more sensitive to the difference in reward probabilities. However, the interaction between reward magnitude and reward probability maintained significant positive effect on choice ($\beta_{{M:W}_{\Delta}}=0.05$, *p* = 7.8$\times$10^-9^), and there was no significant two-way interaction between order, reward magnitude, and reward probability ($\beta_{{O:M:W}_{\Delta}}=-0.01$, *p* = .23). In line with these results of logistic regression, the estimated difference parameter for inverse temperature ($D_{\beta}$) did not show significant difference depending on the order of conditions (*t*(195.8)=$-$0.43, *p*=.66, Fig. S3). These results indicate that the differences observed between the two conditions are robust even if we considered the effects of the order of conditions.


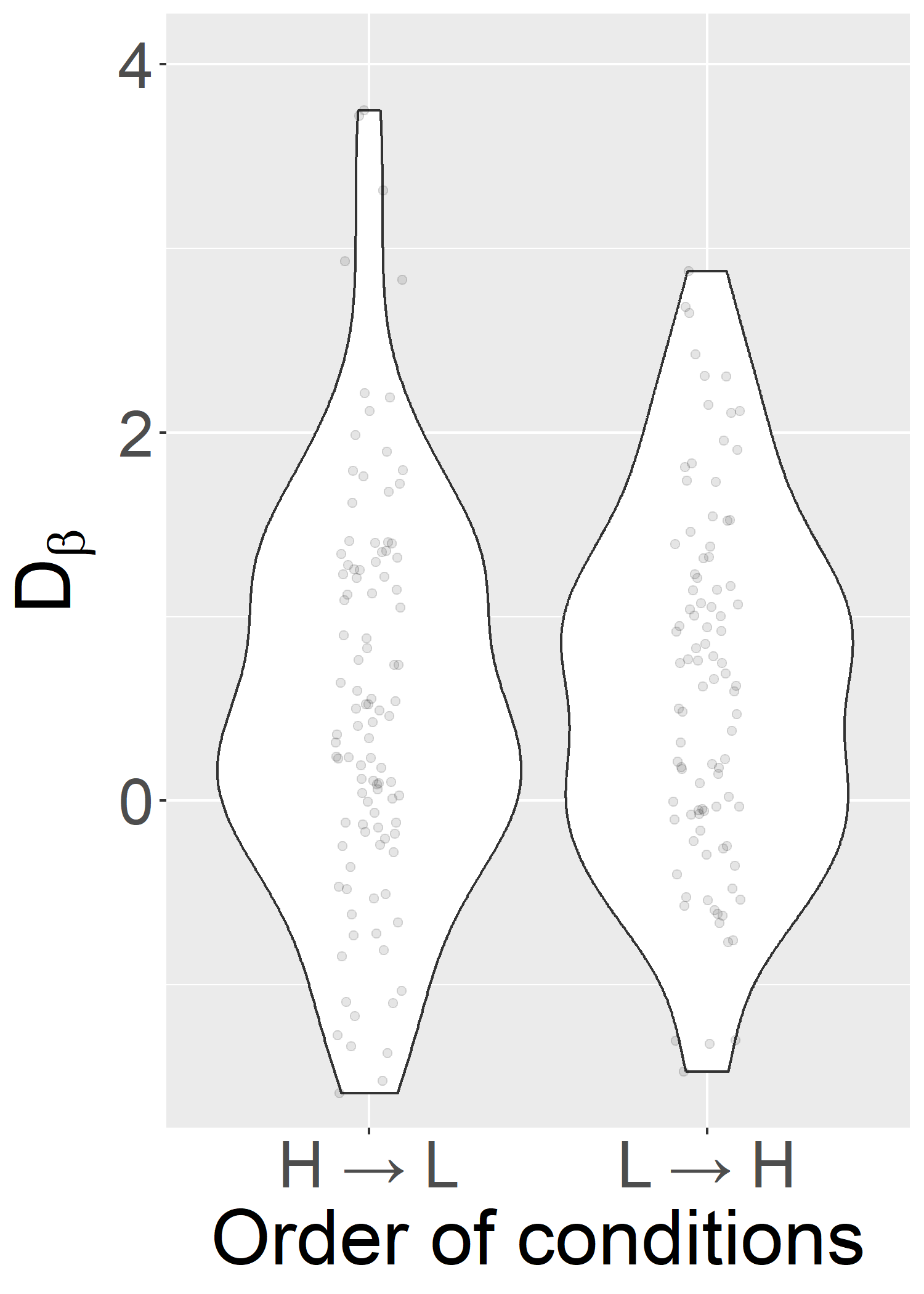


**Fig. S3** The difference parameter of inverse temperature ($D_{\beta}$) stratified by the order of conditions. “H $\to$ L” means those who experienced the high reward condition first, and “L $\to$ H” means those who experienced the low reward condition first.

***The effect of the forgetting rate parameter***

We observed a significant negative interaction between task horizon and expected reward probability on choice. This apparently odd result is indicated to be a byproduct of forgetting effect and does not suggest that participants were becoming less exploitative as the trials progress (Cockburn et al., 2022). As the forgetting rate increases, the weighting of more recent observations becomes stronger, leading to greater discrepancies from the optimal value estimates assumed in the logistic regression. This deviation is thought to cause the apparent negative interaction between task horizon and expected win probability (see Figure S1 of Cockburn et al. (2022) for details). In fact, when we simulated the behavior of a model without forgetting (i.e., $\eta=0$), we observed no decrease in the proportion of choosing options with higher expected win probability (Fig. S4), unlike what was observed with real data (Fig. 4a) and models with forgetting (Fig. S3).


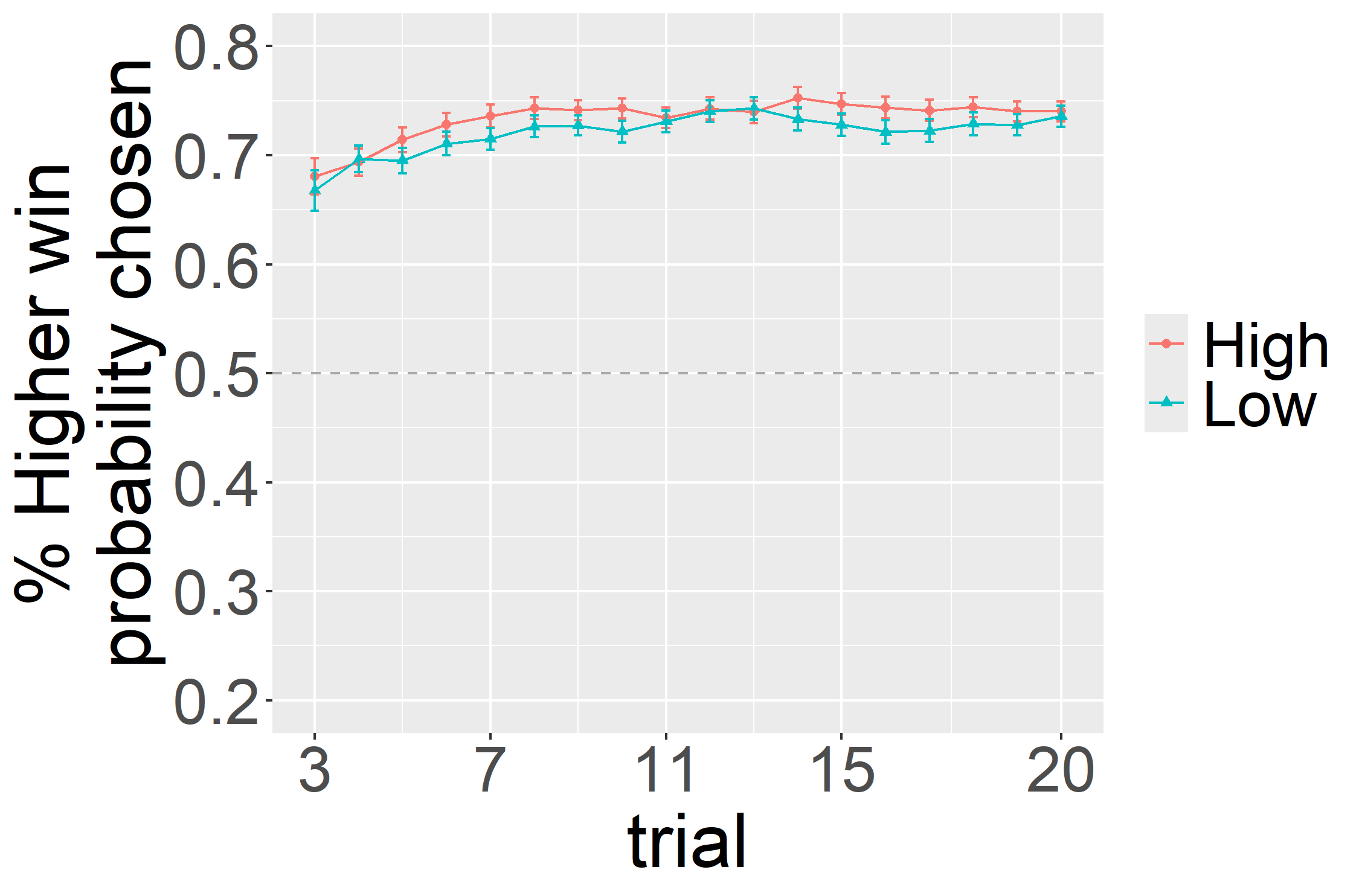


**Fig. S4** The effect of interaction between task horizon and expected win probability on choice in the simulated data of “novelty-biased familiarity-gated uncertainty model” without the forgetting rate parameter. The magenta circles and line represent the result of the high reward condition and the cyan circles and line represent the result of the result of the low reward condition. We fitted novelty-biased familiarity-gated uncertainty model without the forgetting rate parameter to actual choice data of participants and estimated parameters for each individual. We then used those estimated parameters to simulate the behavior of the model.

***The effects of reward probability, uncertainty, and novelty and their between-condition differences in simulated behavioral data***

To examine whether “novelty-biased familiarity-gated uncertainty model”, which was selected as the best model in this study, can accurately predict participants’ actual behavior, we generated simulation data for 198 participants using the parameters estimated by HBI. We then examined the effects of reward probability, uncertainty, and novelty as well as their interaction with reward magnitude by summarizing the data as was done for actual data. As can be seen in Fig. S5, the novelty-biased familiarity-gated uncertainty model was shown to be able to capture the behavioral characteristics of actual participants (see Fig. 3b-d and Fig. 4a-c).


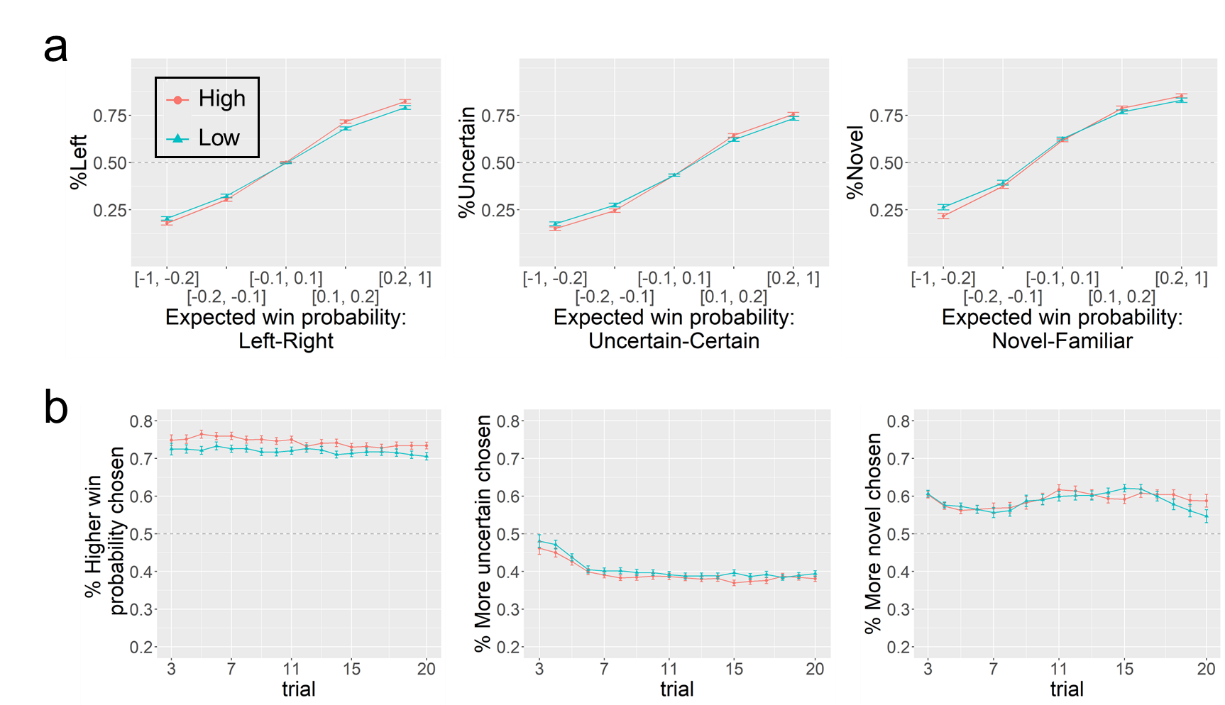


**Fig. S5** The summary of the behavior of 198 simulated agents (“novelty-biased familiarity-gated uncertainty model”) using estimated parameters. (a) Proportion of choosing left stimulus (left), more uncertain option (middle), and more novel option (right), as a function of differences in expected win probability. Magenta rectangles and cyan circles correspond to high and low reward condition, respectively (same applies to (b)). (b) Within-blocks temporal change in a moving average of proportion of choosing the option with higher expected win probability (left), higher uncertainty (middle), and higher novelty (right), averaged across blocks and agents. The time window for the moving average was three trials.

***The relationship between the initial uncertainty bias parameter (U_I_) and performance in our task***

To examine how performance changes depending on the value of $W_{I}$, we simulated how the average number of optimal actions over 300 trials varied when $W_{I}$ took 81 equally spaced values from $-$2 to 2 with the other parameters fixed to the estimated group mean ($\eta$=0.10, $\beta$=5.34, $N$=0.01 , $U_{T}$=$-$0.34).

As shown in Fig. S6, average number of optimal actions had inverted U-shaped relation with $W_{I}$, taking maximal value when $W_{I}$ was close to 0. The group-level estimates of our participants were negative but close to 0 (gray dotted and dashed lines of Fig. S3) in both conditions, which suggests that participants showed nearly optimal initial uncertainty bias in both conditions.


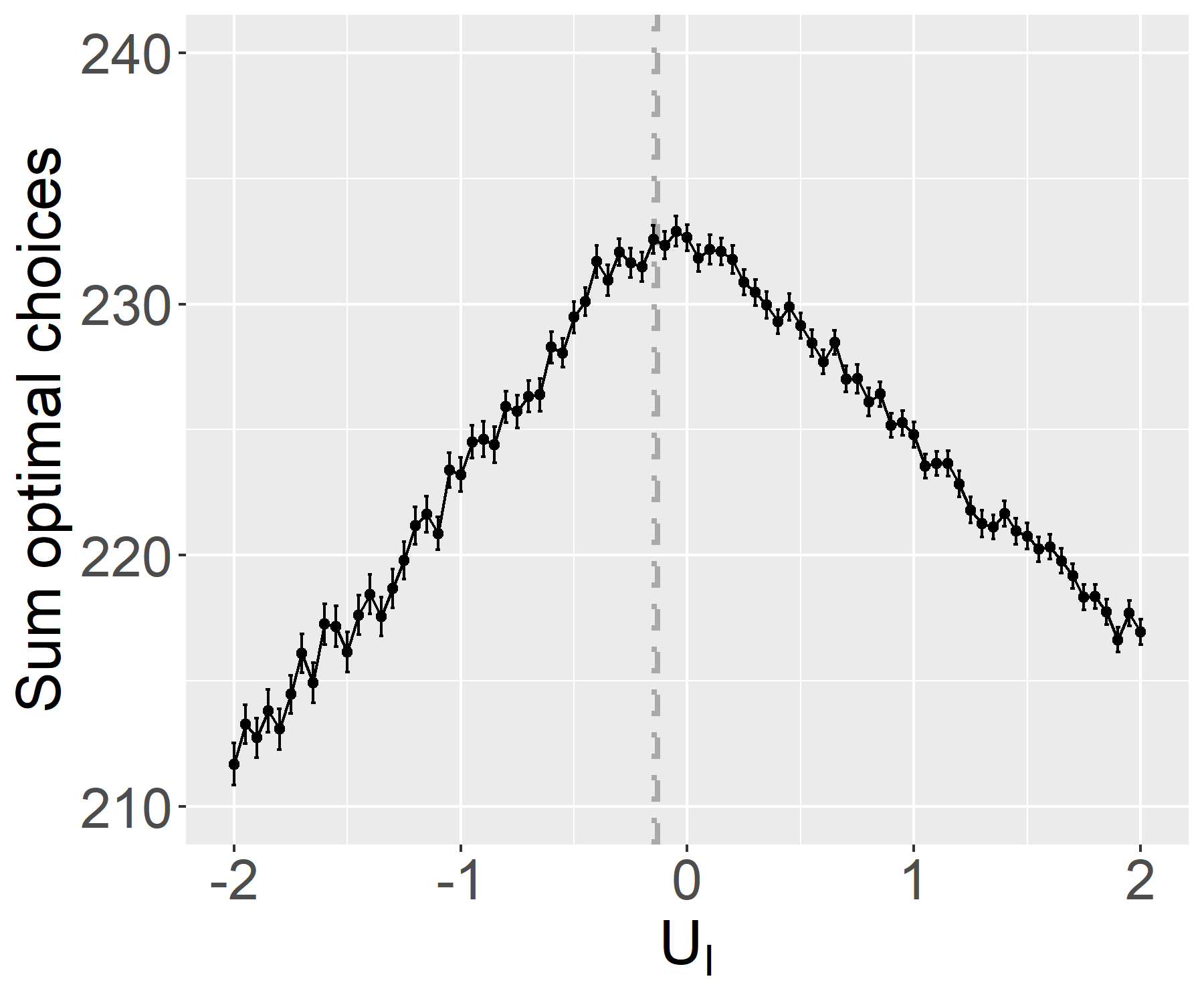


**Fig. S6** The average total number of optimal actions as a function of the initial uncertainty bias parameter ($U_{I}$). Error bars represent standard error of the mean. Gray dotted line and dashed line represents the group-level estimate of $U_{I}$ in high and low reward condition, respectively.

***Comparison of memory test performance in the two conditions***

To test whether memory test performance differed between high and low reward conditions, we conducted linear mixed modeling using memory test accuracy as a dependent variable and condition as an independent variable, assuming the random effect of participant. As stated in the main text, we found that there was no significant fixed effect of condition on performance ($b_{condition}=-0.002, p= .86$; Fig. S7), suggesting that participants engaged in the two conditions with similar levels of motivation.


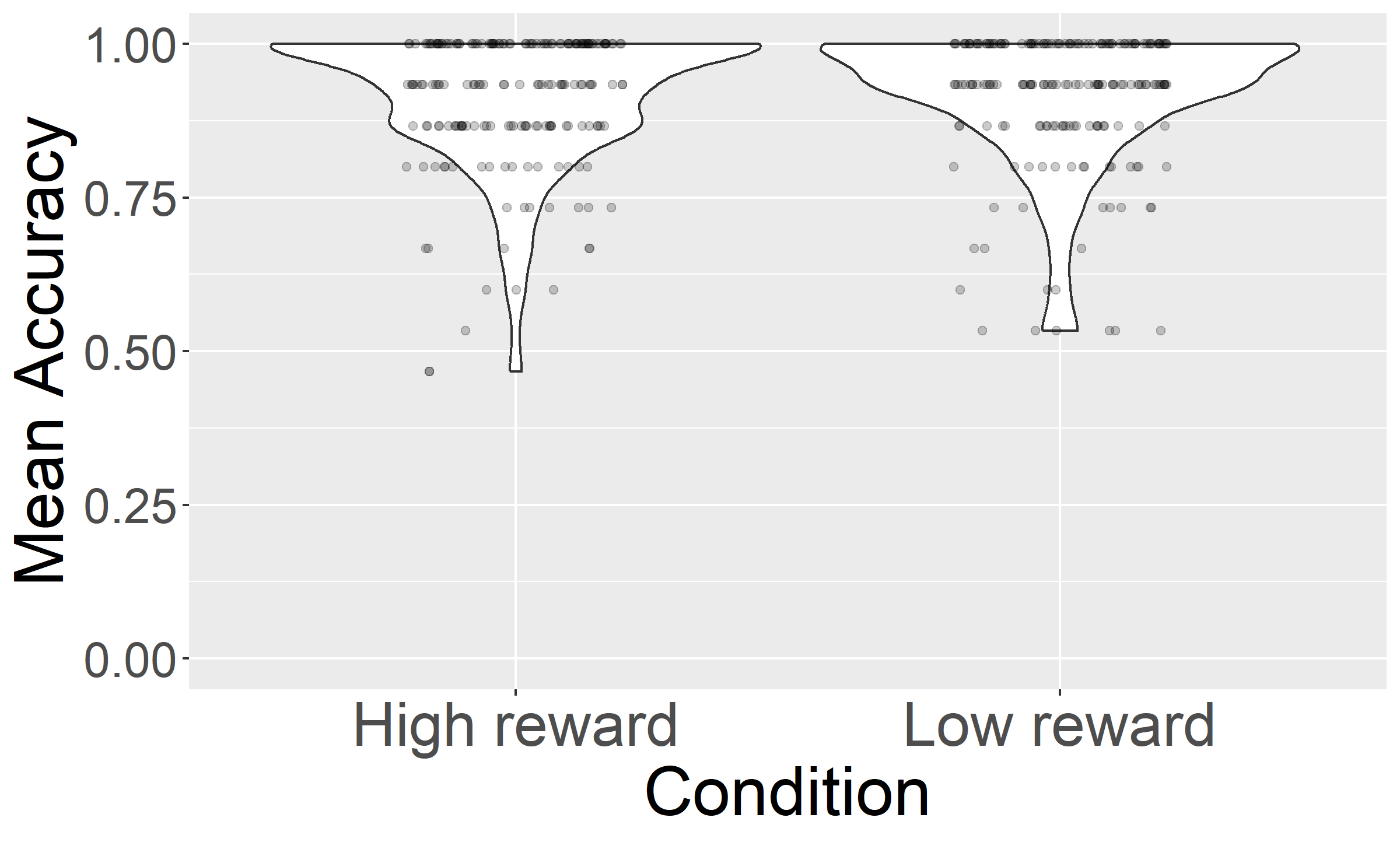


**Fig. S7** Mean memory accuracy of the stimuli used in the high reward condition and low reward condition. There was no significant effect of condition on memory accuracy.
